# The History of Methicillin-Resistant *Staphylococcus aureus* in Brazil

**DOI:** 10.1155/2020/1721936

**Published:** 2020-10-07

**Authors:** Mariana Moreira Andrade, Wilson Barros Luiz, Rayane da Silva Oliveira Souza, Jaime Henrique Amorim

**Affiliations:** ^1^Laboratório de Agentes Infecciosos e Vetores, Centro Das Ciências Biológicas e da Saúde, Universidade Federal Do Oeste da Bahia, Barreiras, Bahia, Brazil; ^2^Programa de Pós-graduação em Biologia e Biotecnologia de Microrganismos, Universidade Estadual de Santa Cruz, Ilhéus, Bahia, Brazil

## Abstract

Since the emergence of MRSA in the 1960s, a gradual increase in infections by resistant bacteria has been observed. Clinical manifestations may vary from brand to critical condition due to host risk factors, as well as pathogen virulence and resistance. The high adaptability and pathogenic profile of MRSA clones contributed to its spread in hospital and community settings. In Brazil, the first MRSA isolates were reported in the late 1980s, and since then different genetic profiles, such as the Brazilian epidemic clone (BEC) and other clones considered a pandemic, became endemic in the Brazilian population. Additionally, Brazil's MRSA clones were shown to be able to transfer genes involved in multidrug resistance and enhanced pathogenic properties. These events contributed to the rise of highly resistant and pathogenic MRSA. In this review, we present the main events which compose the history of MRSA in Brazil, including numbers and locations of isolation, as well as types of staphylococcal cassette chromosome *mec* (SCC*mec*) found in the Brazilian territory.

## 1. Introduction

Outbreaks of nosocomial and community-associated infections with methicillin-resistant *Staphylococcus aureus* (MRSA) have been reported as highly relevant worldwide. Attention to such pathogenic bacteria increased progressively since the first reports of resistance to antimicrobial agents. Penicillin was the first antibiotic to be introduced in clinical practice, in 1940. Shortly after, the selection of *β*-lactamase-producing bacteria marked the beginning of the first wave of antibiotic resistance in *Staphylococcus aureus* (*S. aureus*), which continues today [1].

The rapid spread of penicillin resistance briefly came to a halt after the introduction of the second-generation, semisynthetic methicillin in the 1960s. However, MRSA soon emerged in England, and only in 1981, this mechanism of resistance was unraveled: these strains harbored mutant penicillin-binding proteins, designated PBP-2a, which showed a reduced affinity for methicillin. PBP-2a is encoded by *mecA*, a gene located in the *S. aureus* chromosome [2]. Thereafter, new cases of hospital-acquired infections were reported in other countries such as Australia and the United States [3, 4]. Due to the use of new antibiotics, a slight decrease in MRSA prevalence was noticed. However, because of selective pressure, strains of *S. aureus* began to display a multidrug resistance profile. Cases of MRSA resistant to both *β*-lactams and gentamicin began to be reported in health units at the end of the 1970s [5–7]. In the 1980s, reports of outbreaks and infections caused by MRSA increased gradually.

The genetic profile of MRSA began to be clarified only after 1999, when the gene coding a mutated form of the PBP of the N315 resistant clone isolated in Japan, in 1982, was discovered [8]. In 2001, it was reported that such a sequence was inserted into a mobile cassette within the chromosomal DNA, called staphylococcal cassette chromosome *mec* (SCC*mec*). Thereafter, the first three isolates of SCC*mec*-containing *S. aureus* were used to designate the first three types of cassettes, in order of isolation [9]. To date, fourteen types of SCC*mec* were described in *S. aureus* [10, 11]. They were identified according to different combinations of components of their sequences, including the *mec* complex, the cassette chromosome recombinase (*ccr*) complex, and J regions [10, 12].

Brazil is the largest country in Latin America and the 5^th^ largest country in the world. Reviewing the history of MRSA in Brazil will help to better understand the spread of this important pathogen in Latin America, as well as in the new world. In Brazil, there are some epidemiological surveillance systems of resistant bacteria which do not work at a national level [13, 14]. However, in 2018, a program named PAN-BR (National Action Plan for the Prevention and Control of Antimicrobial Resistance in Brazil) was developed [15]. Although it is not specific for the control and monitoring of MRSA, it was designed based on objectives pre-established by organizations, such as the World Health Organization, and aims to apply strategies for the prevention, control, and monitoring of infections caused by antimicrobial-resistant pathogens, including MRSA. One of the strategic objectives of PAN-BR is “to strengthen knowledge and the scientific basis through surveillance and research” [15]. Therefore, the data provided in this review will contribute to the performance and development of this program, as well as other strategic action plans suggested by the Agência Nacional de Vigilância Sanitária (ANVISA) for the prevention and control of resistance in the country [16].

In this review, we present the history of MRSA in Brazil. Numbers and locations of isolation, as well as types of SCC*mec* found in the Brazilian territory, are discussed in sections by decade, since the 1980s. As inclusion criteria, all published studies reporting the isolation of MRSA from human samples in Brazil were used in this review. In addition, the prevalence of MRSA by region, as well as the frequency types of SCC*mec*, is shown. Text sections are concentrated on a critical review of the main events which compose the history of MRSA in Brazil. The articles were searched in MEDLINE/PubMed and SciELO databases by using the keywords “MRSA Brazil.” We found 597 articles, and after applying exclusion and inclusion criteria, 199 articles were selected.

### 1.1. The 1980s

**Figure 1:**
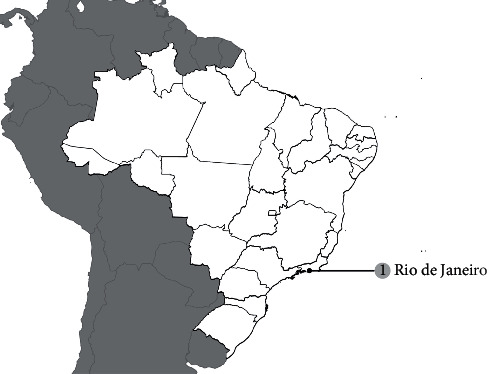
Map with georeference of MRSA reports showing the number of publications by isolation location during the 1980s.

The first cases of MRSA in Brazil were reported in 1987 in Rio de Janeiro, and although they were not published, these events were mentioned by Ramos et al. in 1999 [17]. Such publication was the only one to report MRSA in the 1980s in Brazil, as shown in Figure 1. The incidence of MRSA was reported to be approximately 8%. Variances in the occurrence of MRSA isolates were reported in the following two years: with a decrease of 7.2% in 1988 and the following increase to 33% in 1989 [17]. From 1987 to 1994, Tresoldi and colleagues reported that 257 of 421 *S. aureus* isolates were MRSA. However, although *S. aureus* is to be isolated with the highest frequency (20.9%), MRSA isolates in this study were reported only in the 1990s by Tresoldi and colleagues [18].

After the global spread of MRSA, new antimicrobial agents, such as Synercid, daptomycin, linezolid, and tigecycline, were introduced during the treatment of infections caused by methicillin-resistant bacteria, which may have contributed to broadening mechanisms of multidrug resistance [19–23]. In a study published in 1989, which involved 106 strains of *S. aureus* from 21 countries, including Brazil, 90% of the samples were shown to be multiresistant to antimicrobial agents. Relevantly, the Brazilian strains showed resistance to fourteen antibiotics in this same study [24].

### 1.2. The 1990s

The first three waves of resistance of *S. aureus* of antimicrobial drugs were characterized based on its spread specifically in health care environments. The first wave was characterized by the emergence of strains capable of producing penicillinase, which inactivates penicillin. The emergence of MRSA strains marked the second wave. The disappearance of the archaic clone and the raise of new clones marked the third resistance wave [25]. The fourth wave has been marked by the introduction of community-acquired MRSA (CA-MRSA). However, the hospital clones were still prevalent in Brazil in the 1990s [26]. Compared to the 1980s, the number of occurrences of MRSA increased gradually in different health care facilities in Brazil [17, 27–30]. The spread of MRSA continued to be reported in different hospitals in São Paulo [31, 32]. Restriction fragment length polymorphisms (RFLP) showed the spread of MRSA clones, which indicated that microbial transfer was occurring possibly due to an interhospital connection involving patients and health workers [31, 32]. Furthermore, 91 MRSA isolates were found as microbiota composing of hospital food handlers in Teresina [33]. Such results indicated that transmission by physical contact was a determining factor of the occurrence for nosocomial outbreaks caused by both susceptible and resistant *S. aureus* [34, 35].

The theory of interhospital connection was reinforced when the same clone of MRSA was isolated from different locations at the same hospital in Joao Pessoa, in 1992 [36] and thereafter was found in the Campinas University Hospital, in two different studies [18, 37]. Such a clone was shown to be the same one found by Sader et al. in São Paulo, in 1993 and 1994 [31, 32]. The spread of MRSA was also shown to happen in an intrahospital way, as reported by a study carried out in Rio de Janeiro, in which propagation of a single virulent multidrug-resistant clone within the same hospital caused a relevant number of deaths [38].

A study carried out in five Brazilian cities showed in a systematic way that the interhospital communication was not restricted to nearby areas due to the spreading of bacteria with the same genetic pattern to different regions of the country [39]. Such a genetic pattern was identified by pulsed-field gel electrophoresis (PFGE) of chromosomal DNA. The epidemic MRSA was named as Brazilian epidemic clone (BEC) and became one of the five most-discussed MRSA clones around the world [28, 39]. Epidemiological studies aiming to investigate the spread of BEC were carried out in the 1990s in both Brazil and other countries, such as Portugal, Argentina, Chile, and Italy [28, 40–42]. MRSA strains containing both the polymorphic form type XI of the *mec* gene and type B of the Tn554 gene were classified as the A genetic pattern of BEC (BEC A). Although this pattern has been identified more frequently, some studies have isolated MRSA that differed minimally from BEC A. Such variants, including BEC A, were grouped as the Brazilian epidemic clonal complex (BECC) [30, 43].

After such reports, BEC was massively searched in several hospitals of Brazil evidencing its wide geographical distribution and predominance [42, 44]. The study by Oliveira et al. showed that from 83 MRSA isolated in 14 states in Brazil, 78.3% contained the *mecA* gene with polymorphism XI and Tn554 type B (BEC A). Moreover, isolates were shown to be multiresistant to drugs. These results clearly showed the spread of the BEC and its variants in Brazil [45, 46].

Genetic patterns of BECC isolates were shown to be diverse regarding their antimicrobial resistance and pathogenicity such as to be capable of forming biofilm and adhere and invade airway epithelial cells [30, 47]. The multiresistance is another common feature among BECC isolates. They carry structures such as plasmids and transposons that are responsible for resistance to other drugs within the cassette. In addition to methicillin, strains may be resistant to clindamycin, erythromycin, cephalothin, gentamicin, ciprofloxacin, sulfamethoxazole-trimethoprim, and chloramphenicol [39]. These strains have also shown high-level resistance to mupirocin. It is due to the insertion of the PMG1 plasmid, which carries a novel *ileS* gene that encodes a novel isoleucyl-tRNA synthetase, homolog to antibiotic target [48–50]. Such multiresistance presented by MRSA and the high frequency of nosocomial infections are associated with risk factors such as insertion of classic pathogens into host microbiota, prolonged hospitalization, and misuse of antibiotics [51–53]. This ability to adapt is probably related to genetic diversity into the BECC reported in different published studies [54, 55]. These reports indicated a relevant genetic diversity into the BECC at the ending of the 1990s.

**Figure 2:**
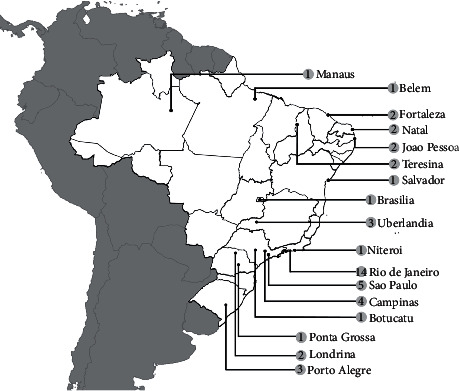
Map with georeference of MRSA reports showing the number of publications by isolation location during the 1990s.

Therefore, other clones or subclones were reported in Brazilian hospitals. The first report of non-BEC MRSA, which presented different genetic patterns from that characterized by BEC, such as *mecA* type III and Tn544 type B polymorphism, was carried out in 1996 [56]. Although there were reports of diversity in genetic profiles, the BEC A was still prevalent among the isolates, as occurred in Teresina, Rio de Janeiro, Uberlandia, and Belem [30, 49, 57, 58]. However, the type of SCC*mec* was not yet known. In the 2000s, it was reported that the BEC clone carries the SCC*mec* type III. Such a cassette type was thus shown to be the most frequent in Brazil in the 1990s [55, 59]. The number of MRSA isolation reports published in the 1990s is shown in Figure 2.

### 1.3. The 2000s

**Figure 3:**
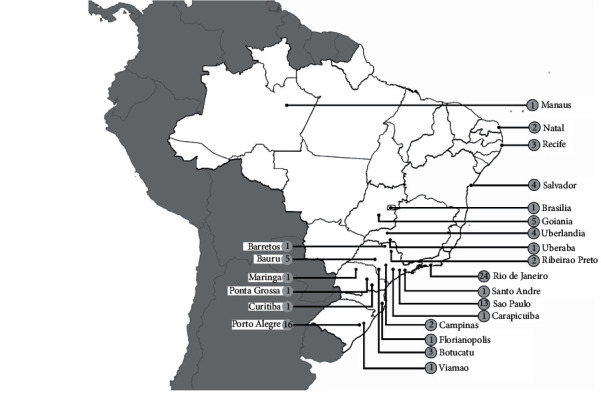
Map with georeference of MRSA reports showing the number of publications by isolation location during the 2000s.

In the 2000s, MRSA remained a concern in Brazil, with new records in the prevalence of resistant bacteria, with an increase in the number of cases of infections [60].  Figure 3 shows the number of reports by location published in this decade. Strains of the BECC continued to be predominant in Brazilian hospitals until a certain moment [29, 61–66]. However, over the decade, other international (non-BEC) clones were imported and started to be reported in different regions of Brazil [67, 68]. Such international clones carried other types of cassettes and were named according to the location they were isolated for the first time [69]. Some of these clones, although considered pandemic, were reported less frequently in Brazil, such as the Iberian clone (SCC*mec* type I); the Hungarian clone (SCC*mec* type III); Cordobes/Chilean clone (SCC*mec* type I); an MRSA clone carrying SCC*mec* type V [69–71]. However, other clones considered to be of great relevance in a global context were reported in a larger frequency. One of these clones was the New York/Japan clone, which is also classified as HA-MRSA (hospital-acquired MRSA) but carrying SCC*mec* type II. Such a clone was initially isolated in a lower frequency with regard to the BEC. Nevertheless, it was gradually spread in the hospital environment [47, 65, 71–73]. Its fixation in Brazilian hospitals was a milestone in history because, in addition to its spread, the New York/Japan clone presented resistance to *β*-lactam antibiotics, ciprofloxacin, erythromycin, and clindamycin, hindering the treatment of patients [74].

The pediatric clone, which is of great world relevance, had also an important role in the history of MRSA in Brazil. It carries the SCC*mec* type IV, which is commonly present in strains of CA-MRSA. However, although not classified as HA-MRSA, it was reported in a relevant number of nosocomial infections. Its profile of resistance diverges from most of HA-MRSA, presenting a susceptibility to a wide range of antimicrobial agents, except *β*-lactams. Although non-multidrug resistant, the pediatric clone began to exhibit important virulence factors. Isolates obtained in different cities showed the capacity of forming biofilm and of producing enterotoxins. Such virulence factors increase the bacterial pathogenicity, exacerbating infections mainly in immunocompromised, children, and elderly [47, 72, 75, 76].

The pediatric clone has similarities and divergences with another clone that also gained prominence: the Oceania Southwest Pacific (OSP) clone. In common, both carry SCC*mec* type IV and are typically non-multiresistant. However, different from the pediatric clone, the OSP clone was shown to cause infections in the community [77]. Community MRSA strains were first reported in western Australia in the early 1990s [78]. Such strains were initially devoid of Panton-Valentine leucocidin (PVL), but subsequent cases of community MRSA were now recorded as PVL positive [79]. Such a clone was first described in Brazil as CA-MRSA before being isolated from patients with skin and soft tissue infections which were not exposed to classical risk factors for nosocomial infection [80]. Several infections caused by CA-MRSA ranging from mild [81–85] to severe [86–88] were reported in Brazil in the 2000s. Although the properties of CA-MRSA appear to make it less aggressive than hospital clones, the OSP clone presents virulence factors involved in the high pathogenicity, such as the PVL, which kills immune cells and induces tissue necrosis [82, 83, 86, 87, 89, 90]. Although originally found in MRSA SCC*mec* type IV, PVL has also been reported in strains with other types of cassettes mostly present in HA-MRSA strains, such as BEC, due to a horizontal transfer of genes [29, 63, 91, and 92]. Such horizontal transference indicates contact between CA and HA-MRSA. Thus, CA-MRSA was identified in health units, and HA-MRSA clones were identified in the community [74, 93, and 94]. As a result, in addition to the presence of PVL in clones that at the time were considered unusual carriers, the ability to form biofilms was spread among the various types of MRSA [92, 95]. In addition to the spread of virulence factors, genes of resistance to antimicrobial drugs were transferred to non-multiresistant clones, which resulted in strains with a profile of pathogenicity and resistance [75, 89, and 96].

The spread of different clones was observed in the mid-2000s in Brazil [92, 97]. Different clones of MRSA carrying SCC*mec* type IV, including CA-MRSA, were detected in 19 of 20 MRSA isolated from patients with nosocomial infections in a hospital of Rio de Janeiro. In Porto Alegre, moreover to the isolation of the OSP clone, the pediatric clone was shown to be circulating [71, 96, 98, and 99]. In São Paulo, a profile similar to CA-MRSA was identified as the cause of 95% of bloodstream infections. Such reports indicated the adaptive capacity of CA-MRSA to the hospital environment [75].

An important study was published in 2012 describing samples collected in the south, southeast, and northeast of the country and revealed the most frequent cassettes circulating in Brazil. The cassettes were identified based on the detection of clonal complexes (CC) and the most common were shown to be SCC*mec* types II, III, and IV [47, 64, 65, 72, 100, and 101]. These cassettes were reported gradually during the decade of 2000, showing that there was an evolution, adaptation, and propagation of different clones of MRSA in Brazil.

### 1.4. The 2010s

**Figure 4:**
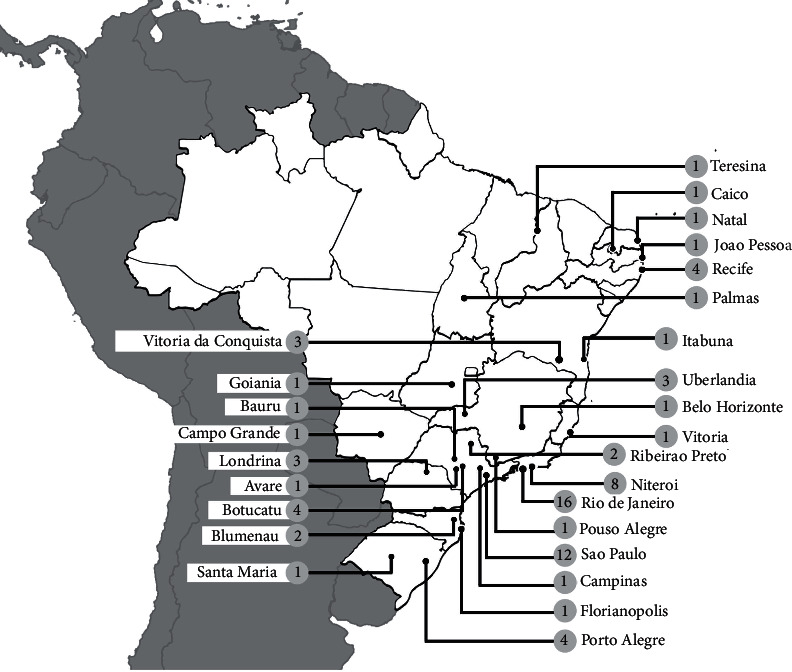
Map with georeference of MRSA reports showing the number of publications by isolation location during the 2010s.

In the 2010s, MRSA remained being increasingly reported in Brazil, as shown in Figure 4. The most common clones in the hospital environment continued to be those carrying the SCC*mec* types II, III, and IV [14, 65, 73, 102, 103]. However, SCC*mec* type II, in contrast to the last decade, was reported as one of the most prevalent clone [103–109]. Relevantly, the New York/Japan clone, which carries SCC*mec* type II, was shown to become resistant to daptomycin, tigecycline, and vancomycin [67, 110]. In addition, such a clone was shown to become capable of producing *α*-hemolysin and PVL and forming a biofilm [14, 104, 105]. Thus, multiresistance and virulence remained evolutionary events among MRSA clones. As a further example of adaptation and evolution, in 2011, a CA-MRSA strain was shown to have acquired the *vanA* gene, which confers resistance to vancomycin. This was the first CA-MRSA reported being resistant to this antimicrobial agent [111].

Clones carrying the SCC*mec* type IV continued to be the main producers of PVL and biofilms [112, 113]. These virulence factors were involved in infections reported in both hospital and community environments due to a horizontal transfer of genes among CA-MRSA and HA-MRSA strains. In addition to the New York/Japan clone, the production of biofilms and PVL began to be seen in more unusual and less frequent clones, such as those carrying SCC*mec* types V and I [114, 115]. These types of cassettes, as well as the SCC*mec* type VI, UK/EMRSA-3, Hungarian, and Iberian clones, were identified in the history of MRSA in Brazil at a low frequency [104, 116–119].

The last relevant chapter of the history of MRSA in Brazil also involves SCC*mec* V. An unusual genetic profile called clonal complex 398 (CC398) began to be reported. Such variants may carry either type IV or type V cassettes. This complex is directly associated with livestock and thus called LA-MRSA (livestock-associated methicillin-resistant *Staphylococcus aureus*). This type of MRSA emerged the first time from animal infection samples, in 1972 [120], but was subsequently isolated from humans, especially those who had direct access to animals [121–123]. In 2010, it was first isolated in Brazil from a patient with cystic fibrosis who had contact with animals from the farm [124]. Later, between 2011 and 2016, six clones of the same complex were isolated from children in Rio de Janeiro [125]. However, such isolates were not classified as LA-MRSA because the children did not present the typical risk factors for the acquisition of this lineage, which includes the previous contact with animals. These results showed that clones of this lineage are not restricted to animals and have adapted to a new kind of host.

### 1.5. Clinical and Epidemiological Relevance

The 199 documented articles reflect the high and gradual incidence of pandemic clones disseminated in Brazil and the increase in the proportion of infections that result in different clinical manifestations. Although it is currently part of the PAN-BR, no survey has yet been made of the MRSA rates recorded since its emergence in Brazil until today [15]. However, Brazil is part of an antimicrobial surveillance organization that acts at a global level, called SENTRY, which recorded that 38.7% of MRSA out of 17474 samples of *S. aureus* collected over the 20-year interval were from Latin America, including Brazilian sampling [126].

**Figure 5:**
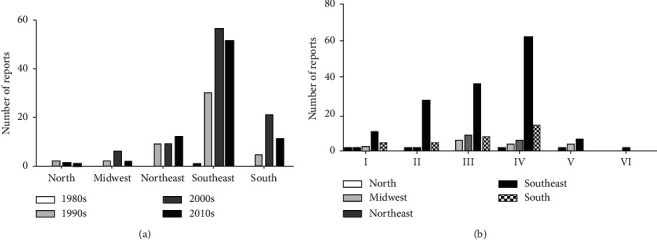
MRSA isolates and the types of *SCCmec* reported in Brazil. (a) Numbers of MRSA isolations per decade in the five regions of Brazil. (b) Numbers of types of SCC*mec* reported in the five regions of Brazil until 2019. The graphics program used to create the figure was GraphPad Prism.

A quantitative survey of reports of MRSA infections specifically in Brazil collaborates to point out how emergency it is to apply the objectives of national epidemiological inspection and control programs [15]. Such a survey based on the regions of Brazil evidences which areas are most affected and which increase the country's epidemiological rates.  Table 1 shows all the cities in Brazil and its respective references that were reported through publications that involved isolating MRSA from human samples. It is evident that the spread of clones and the consequences they bring to patients were gradually increased over the years and that all regions of the country have already been notified, with the southeast region being the most affected in all decades, followed by the south and northeast (Figure 5(a)). The high number of MRSA notifications in these regions was probably due to the fact that they are the most populated in Brazil, while the centralwest and north regions have lower demographic density [236]. In all regions, SCC*mec* type IV is the most prevalent, followed by III, II, and I, demonstrating that the clones that typically circulate in the community are the most prevalent in the country, followed by hospital clones (Figure 5(b)). Such data show how these virulent, highly adaptable, and multidrug-resistant clones pose risks to the population and show that such a survey contributes to the development of prevention and control assistance plans recently adopted in the country.

### 1.6. Final Considerations

Considering case reports and field research included in this review, it is clear that MRSA is now present in the five regions of Brazil. It is also clear that Brazil has a large genetic diversity of MRSA, including multidrug-resistant and high virulent strains. Such diversity may increase if new SCC*mec* and variants are imported. In addition, it is important to note that the data indicated in this review are relevant but still limited concerning the subcontinental size of Brazil. Limitations may include an insufficient number of studies due to low government investment in research, lack of access to health services for the vulnerable population, and application of empirical antibiotic therapy ignoring established protocols, which undoubtedly results in underreporting of a relevant number of cases. This review reinforces problems related to the ability of bacteria to become resistant to antibiotics and their potential for spread, usually occurring in epidemic waves initiated by one or a few successful clones. Moreover, it contributes to an epidemiological study by mapping the spread of MRSA in Brazil, as there is still no monitoring system for these resistant strains or a specific antimicrobial surveillance system in Brazil. Strategies of control and monitoring should be increased in hospital and community environments to avoid the advance of spreading of successful clones as well as exporting or importing new strains of MRSA to Brazil.

## Figures and Tables

**Table 1:** Reports of isolation of MRSA from the 1980s to the 2010s in Brazil.

Region/location	1980s		1990s		2000s		2010s	
	*n* ^a^	Refs^b^	*n*	Refs	*n*	Refs	*n*	Refs
North								
Belem	NR^c^	NR	1	[30]	NR	NR	NR	NR
Manaus	NR	NR	1	[39]	1	[81]	NR	NR
Palmas	NR	NR	NR	NR	NR	NR	1	[127]

Northeast								
Joao Pessoa	NR	NR	2	[27, 36]	NR	NR	1	[128]
Itabuna	NR	NR	NR	NR	NR	NR	1	[129]
Salvador	NR	NR	1	[130]	4	[60, 84, 131, 132]	NR	NR
Vitoria da Conquista	NR	NR	NR	NR	NR	NR	3	[102, 114, 133]
Caico	NR	NR	NR	NR	NR	NR	1	[134]
Natal	NR	NR	2	[135, 136]	2	[137, 138]	1	[139]
Teresina	NR	NR	2	[33, 57]	NR	NR	1	[140]
Recife	NR	NR	NR	NR	3	[47, 141, 142]	4	[118, 143–145]
Fortaleza	NR	NR	2	[135, 136]	NR	NR	NR	NR

South								
Viamao	NR	NR	NR	NR	1	[146]	NR	NR
Porto Alegre	NR	NR	3	[39, 147, 148]	16	[61, 69, 80, 82, 83, 88, 89, 98, 146, 149–155]	4	[156–159]
Santa Maria	NR	NR	NR	NR	NR	NR	1	[160]
Ponta Grossa	NR	NR	1	[161]	1	[161]	NR	NR
Maringa	NR	NR	NR	NR	1	[77]	NR	NR
Londrina	NR	NR	2	[135, 136]	NR	NR	3	[104, 105, 162]
Blumenau	NR	NR	NR	NR	NR	NR	2	[110, 117]
Florianopolis	NR	NR	NR	NR	1	[154]	1	[117]
Curitiba	NR	NR	NR	NR	1	[163]	NR	NR

Southeast								
Rio de Janeiro	1	17	14	[17, 28, 29, 34, 38, 39, 48–50, 53, 54, 56, 135, 136]	24	[29, 47, 64–66, 73, 83, 86, 87, 90–92, 95, 96, 99, 124, 151, 164–170]	16	[103, 112, 113, 115, 116, 125, 171–180]
Niteroi	NR	NR	1	[39]	NR	NR	8	[125, 181–187]
São Paulo	NR	NR	5	[31, 32, 39, 42, 188]	13	[72, 75, 85, 93, 151, 154, 189–195]	12	[106, 107, 109, 111, 119, 186, 196–201]
Carapicuiba	NR	NR	NR	NR	1	[202]	NR	NR
Barretos	NR	NR	NR	NR	1	[203]	NR	NR
Bauru	NR	NR	NR	NR	5	[63, 101, 204–206]	1	[108]
Ribeirao Preto	NR	NR	NR	NR	2	[97, 207]	2	[208, 209]
Botucatu	NR	NR	1	[55]	3	[62, 71, 210]	4	[14, 211–213]
Avare	NR	NR	NR	NR	NR	NR	1	[214]
Santo Andre	NR	NR	NR	NR	1	[215]	NR	NR
Campinas	NR	NR	4	[18, 37, 44, 216]	2	[97, 217]	1	[218]
Pouso Alegre	NR	NR	NR	NR	NR	NR	1	[219]
Uberaba	NR	NR	NR	NR	1	[220]	NR	NR
Uberlandia	NR	NR	3	[49, 50, 52]	4	[221–224]	3	[225–227]
Belo Horizonte	NR	NR	NR	NR	NR	NR	1	[67]
Vitoria	NR	NR	NR	NR	NR	NR	1	[228]

Midwest								
Campo Grande	NR	NR	NR	NR	NR	NR	1	[229]
Goiania	NR	NR	NR	NR	5	[70, 76, 94, 230, 231]	1	[232]
Brasilia	NR	NR	1	[135, 136]	1	[154]	NR	NR

*Unknown*	NR	NR	4	[51, 58, 233, 234]	3	[100, 193, 235]	NR	NR

^a^Numbers of reports, ^b^references, ^c^nonreported (NR).
